# Case of Rupture of the Bladder

**Published:** 1847-03

**Authors:** 


*Case of Rupture of the Bladder.*—Mr. Chaldecott records, in a late number of the Provincial Journal, an interesting case of this form of injury. The facts seem too clear to admit of doubt as to the nature of the case. The following is an outline of the history:

A healthy and temperate man, of about fifty, having passed two or three hours at a concert, ran across the street to empty his distended bladder, at twelve o'clock at night; and the night being dark, he did not see a newly erected post, with the top of which the lower part of his abdomen came in violent contact. He states that he fell, and with great difficulty reached his home, which was about a hundred yards distant.

Mr. Chaldecott saw him about half an hour after the accident. He was faint, and suffering severe pain over the abdomen, with desire, but no power to pass his urine. A full-sized catheter was passed easily and completely into the bladder, but no urine escaped. He was placed in bed, and hot fomentations were used to the belly until reaction took place, with which came increase of pain. Twenty leeches were applied, and a gum catheter, but with the same unsatisfactory result as before, not a drop of urine escaping through it.

Mr. Key being sent for, he arrived about eighteen hours after the accident, by which time the symptoms of peritonitis had increased to an alarming degree. The belly was painful, swollen, and tender; the pulse rapid and feeble; and the countenance anxious. Mr. Key passed a catheter, (none having been used for the previous four hours,) and about an ounce of bloody urine came through the instrument. Mr. Key concurred in opinion as to the nature of the injury, and the nearly hopeless prospect for the patient.

At ten o'clock, he had two scruples of liquor opii sedativus, which, after a few hours, produced comfortable sleep; and about four hours from Mr. Key's visit, Mr. Chaldecott passed the catheter, and drew off about four ounces of clear urine. From this time, the pain, swelling, and heat in the stomach and abdomen, gradually lessened, and it was evident that the bladder now held, as on each introduction the catheter brought away clear urine.

From this time until the 13th, (that is, the sixth day from that on which the accident happened,) all went on well, excepting that a smart attack of gout occurred on the 10th, although the patient had never before suffered one ; but on the 13th, from a strong desire to become independent of the catheter, he made straining efforts to pass his water, and he had scarcely passed a tablespoonful, when he felt (to use his own expression) something give way, and a burning pain all over his stomach and bowels, as if boiling water had been poured over them, and the same symptoms of faintness and distress occurred as when the accident first happened.

Mr. Chaldecott saw him within a few minutes of this reopening of the wound of his bladder, for such, no doubt, had been the consequence of his attempts to pass his water. On using the catheter, not more than a teaspoonful came through the tube. He had now again the symptoms of peritonitis, with the addition of incessant sickness. The same plan of treatment was again adopted, viz., fomentations, leeches, and a full opiate, with calomel.

About four hours after, on the introduction of the catheter, the bladder was again found to retain the urine ; and although the peritonitis had increased to a severe degree, the pain, tenderness, and sickness, gradually subsided ; and by a patient submission to the continued use of the catheter for a fortnight, no more interruption to the patient's amendment occurred, excepting that the gout, which, under the use of colchicum, had nearly disappeared, again became severe, no doubt from the fresh absorption of urine which this second accident had permitted to escape into the cavity of the peritonæum. But by this time it was presumed that the wound in the bladder had closed with sufficient firmness to allow the patient to yield to the desire to evacuate his urine without any straining efforts.

Two months have transpired since the commencement of the case, and he feels no other inconvenience from his accident excepting a dragging sensation over the abdomen, chiefly on the right side, which is much increased when he attempts to lie upon his left side.—*London Lancet.*

